# Molecular Blocking of CD23 Supports Its Role in the Pathogenesis of Arthritis

**DOI:** 10.1371/journal.pone.0004834

**Published:** 2009-03-12

**Authors:** Jérôme Rambert, Maria Mamani-Matsuda, Daniel Moynet, Pierre Dubus, Vanessa Desplat, Tina Kauss, Joël Dehais, Thierry Schaeverbeke, Khaled Ezzedine, Denis Malvy, Philippe Vincendeau, M. Djavad Mossalayi

**Affiliations:** 1 Groupe «Thérapeutiques d'inflammation et d'infection», Laboratoire d'Immunologie et de Parasitologie, UFR Sciences Pharmaceutiques, Université Bordeaux 2, Bordeaux, France; 2 Histologie et Pathologie Moléculaire des tumeurs, EA 2406, Université Bordeaux 2, Bordeaux, France; 3 Service de Rhumatologie, Centre Hospitalo-Universitaire Pellegrin, Bordeaux, France; LMU University of Munich, Germany

## Abstract

**Background:**

CD23 is a differentiation/activation antigen expressed by a variety of hematopoietic and epithelial cells. It can also be detected in soluble forms in biological fluids. Initially known as the low-affinity receptor for immunoglobulin E (FcεRII), CD23 displays various other physiologic ligands such as CD21, CD11b/c, CD47-vitronectin, and mannose-containing proteins. CD23 mediates numerous immune responses by enhancing IgE-specific antigen presentation, regulating IgE synthesis, influencing cell differentiation and growth of both B- and T-cells. CD23-crosslinking promotes the secretion of pro-inflammatory mediators from human monocytes/macrophages, eosinophils and epithelial cells. Increased CD23 expression is found in patients during allergic reactions and rheumatoid arthritis while its physiopathologic role in these diseases remains to be clarified.

**Methodology/Principal Findings:**

We previously generated heptapeptidic countrestructures of human CD23. Based on *in vitro* studies on healthy and arthritic patients' cells, we showed that CD23-specific peptide addition to human macrophages greatly diminished the transcription of genes encoding inflammatory cytokines. This was also confirmed by significant reduction of mediator levels in cell supernatants. We also show that CD23 peptide decreased IgE-mediated activation of both human and rat CD23^+^ macrophages. *In vivo* studies in rat model of arthritis showed that CD23-blocking peptide ameliorates clinical scores and prevent bone destruction in a dose dependent manner. *Ex-vivo* analysis of rat macrophages further confirmed the inhibitory effect of peptides on their activation. Taken together our results support the role of CD23 activation and subsequent inflammatory response in arthritis.

**Conclusion:**

CD23-blocking peptide (p30A) prevents the activation of monocytes/macrophages without cell toxicity. Thus, targeting CD23 by antagonistic peptide decreases inflammatory markers and may have clinical value in the treatment of human arthritis and allergic reactions involving CD23.

## Introduction

CD23 has been initially identified as low affinity receptor for IgE on B cells, monocytes, follicular dendritic cells, Langerhan's cells, eosinophils, epithelial cells and platelets. CD23 is distinguished structurally from almost all other immunoglobulin receptors as it belongs to the C-type (calcium dependent) lectin superfamily [1]. In the membrane-bound form of CD23, three lectin domains are spaced from the membrane by a triple α-helical sequence susceptible to proteolysis, leading to the release of various soluble fragments (sCD23) that have oligomeric state-dependent activity [1]. The principal endogenous protease that releases soluble CD23 has recently been identified as ADAM10 (disintegrin-metalloproteinase 10) [2]. CD23 has two isoforms with two splice variants in their first seven (CD23a) or six (CD23b) amino-acid residues in their N-terminal intracellular sequence. The region that differs between the two splice forms is responsible for their distinct signaling activities [3], [4]. They are differentially expressed: CD23a is expressed by antigen-activated B cells before differentiation into antibody-secreting plasma cells, whereas CD23b expression is induced by interleukin-4 (IL-4) on B cells and a variety of inflammatory cells including epithelial cells.

CD23 plays a critical role during immune response including IgE-synthesis, B- and T-cell differentiation, and the secretion of inflammation mediators by various human cells [1]. Cross-linking of surface CD23 promotes the generation of IL-1, IL-6, TNF-α, H_2_O_2_ and nitric oxide synthase-2 (NOS2)-mediated NO through NFκB- and AP-1-dependent mechanisms [5], [6], [7], [8], [9]. We and others have also demonstrated the role of CD23 during inflammation in both Th1 and Th2 response [1], [7], [9]. In addition, soluble CD23 fragments mediate cell activation through the ligation of surface CD11b/c on macrophages [10], [11], or CD21 on lymphocytes [12]. CD23 role during inflammatory diseases was first suggested in rheumatoid arthritis by the ability of anti-mouse CD23 antibody to decrease cellular infiltration of the synovial sublining layer and the destruction of cartilage, in mice with collagen-induced arthritis [13]. Accordingly, CD23-deficient mice had delayed onset and reduced severity of collagen-induced arthritis [14]. Furthermore, CD23 is expressed by peripheral blood monocytes from systemic juvenile arthritis [15] or during adult arthritic crisis, while the physiopathologic role of CD23 remains to be clarified in these diseases. Thus, targeting CD23 could be of interest in the treatment of allergy and inflammatory disorders. Hence, therapeutic CD23 monoclonal antibody (McAb) was recently developed to block human CD23 during chronic lymphocytic leukemia [9].

In the present study, using small synthetic anti-CD23 heptapeptide, we selectively blocked human and rat CD23-mediated cell activation, gene transcription and the generation of various inflammatory factors as to identify new treatment for inflammatory diseases.

## Results

### High affinity of peptide-CD23 interaction

**Figure 1:**
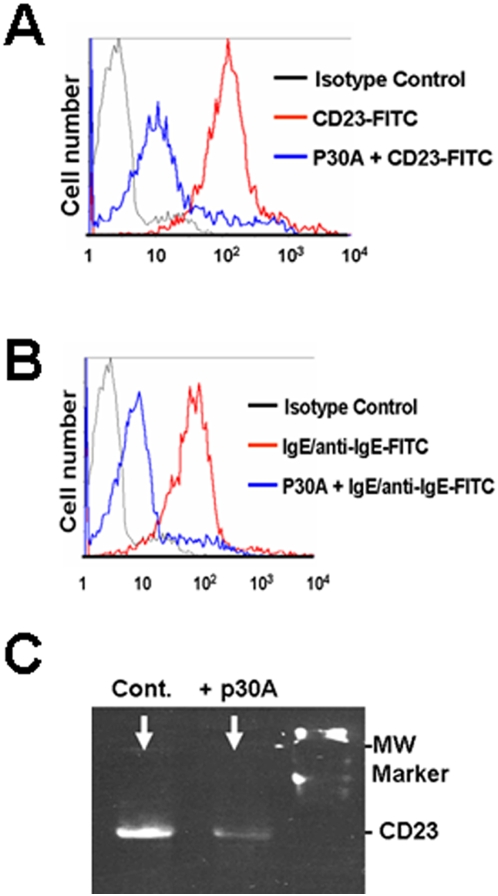
Specific blockade of CD23 reactivity by p30A. Flow cytometry analysis showing the inhibition of (A) anti-CD23-FITC (20 µg/ml) or (B) IgE (10 µg/ml)/anti-IgE-FITC (1 µg/ml) binding to CD23^+^ human PBL-derived macrophages (red lines) or following their pre-incubation with p30A peptides (10 µg/ml) (blue lines). (C) CD23 western blot was performed on total protein extract from à CD23^+^ B-cell line. The detection with CD23-McAb followed the pre-incubation of the blot with or without p30A (10 µg/ml). Similar protein quantity (50 µg) was used for each western blot condition.

Synthetic anti-CD23 heptapeptide p30A (FHENWPS) was obtained using phage display technology as described (see under Materials and methods and PCT Patent 098435). The addition of p30A to normal human macrophages inhibits the binding of FITC-conjugated anti-CD23 McAb (Figure 1A). *In vitro*, p30A was found to block, on the one hand, the binding of IgE/anti-IgE-FITC to human CD23^+^ macrophages (Figure 1B), and on the other hand, the detection of CD23 protein from human CD23^+^ B cell line extract (Figure 1C). We did not observed significant variations of cell mortality and apoptosis in the presence of p30A compared to cells incubated in medium alone (<5%). As p30A was generated against soluble CD23, these data provide strong evidence that p30A interacts with both soluble and surface CD23 forms with a higher affinity than specific McAb or IgE-anti-IgE immune complexes.

### p30A inhibits CD23-mediated cell signaling

**Figure 2:**
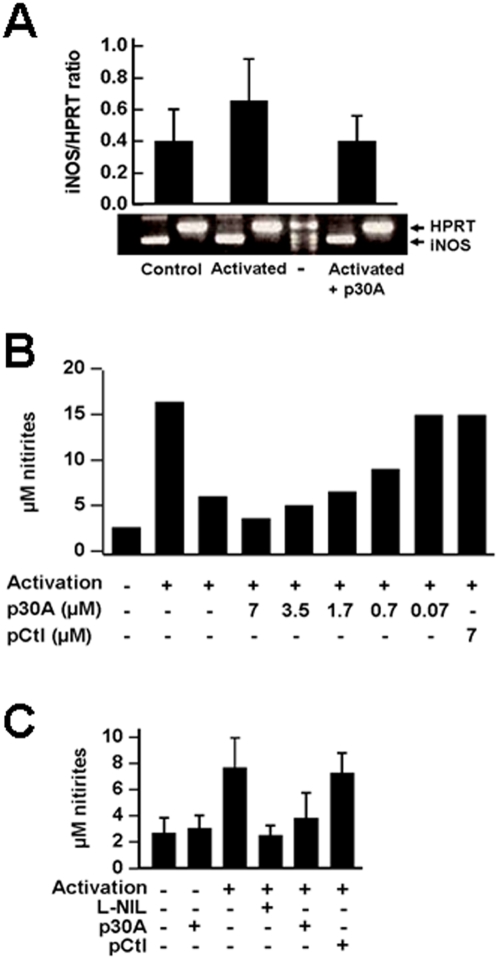
Inhibition of CD23-mediated activation of iNOS pathway in human macrophages by p30A. Human CD23^+^ monocyte-derived macrophages were incubated with cross-linking CD23-McAb (20 µg/ml) alone or following 1 h pre-incubation with 10 µg/ml of various peptides. (A) Inducible NO-synthase expression promoted by CD23-mediated activation is decreased in cells pre-incubated with p30A. Expression of iNOS mRNA was compared to HPRT mRNA levels. (B) Inhibition of NO generation from human activated macrophages by synthetic peptides. Cell supernatants were collected 72 h following cell cultures and levels of nitrites were determined. Dose-dependent reduction of nitrites was obtained with p30A synthetic, whereas control peptides (pCtl) had no effect. Results are expressed as mean of 3 distinct donor cell preparations (SEM<20%). (C) Inducible NOS-mediated NO generation in human macrophages is decreased following pre-incubation with p30A or L-NIL prior to CD23-mediated activation. Results are expressed as mean±s.d. of 7 distinct donor cell preparations.

Upon acquisition of CD23 surface expression, human cells may be activated using its biological ligands (*e.g.* IgE/antigen) or cross-linking with a specific anti-CD23 McAb [8], [16]. McAbs (clones 135 and DM2, IgG1κ) bind human CD23 with high affinity, promote CD23 cross-linking, cell signaling and the transcription of inducible nitric oxide synthase gene [17]. To confirm the ability of p30A to inhibit CD23-mediated signaling in human macrophages, we pretreated cells with purified peptides prior to their activation by McAb. Synthetic p30A reduced the levels of inducible nitric oxide synthase (iNOS)-encoding mRNA (Figure 2A). Nitrites, final metabolites of NO, were measured in cell supernatants as a marker of iNOS activity. Generation of NO from human macrophages was reversed by p30A in a dose-dependent manner (Figure 2B) and similar to N(6)-(1-iminoethyl)-L-lysine/2HCl (L-NIL)(Figure 2C), a specific inhibitor of iNOS [18]. These effects were not observed with an irrelevant heptapeptide, pCtl, used as control (SFNYNYA) (Figure 2B, 2C).

### p30A decreases inflammatory response of normal and arthritic patients' monocyte/macrophage

**Figure 3:**
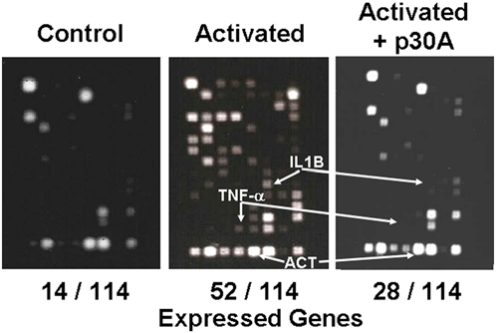
Inhibition of inflammatory gene transcription by p30A in CD23-stimulated macrophages. Human CD23^+^ monocyte-derived macrophages were incubated with cross-linking CD23-McAb (20 µg/ml) alone or following 1 h pre-incubation with 10 µg/ml of p30A. Controls, activated cells with/without p30A pre-incubation are figured. IL-1β, TNF-α and actin (ACT) mRNA location are indicated by arrows. Shown is one representative experiment out of three.

**Figure 4:**
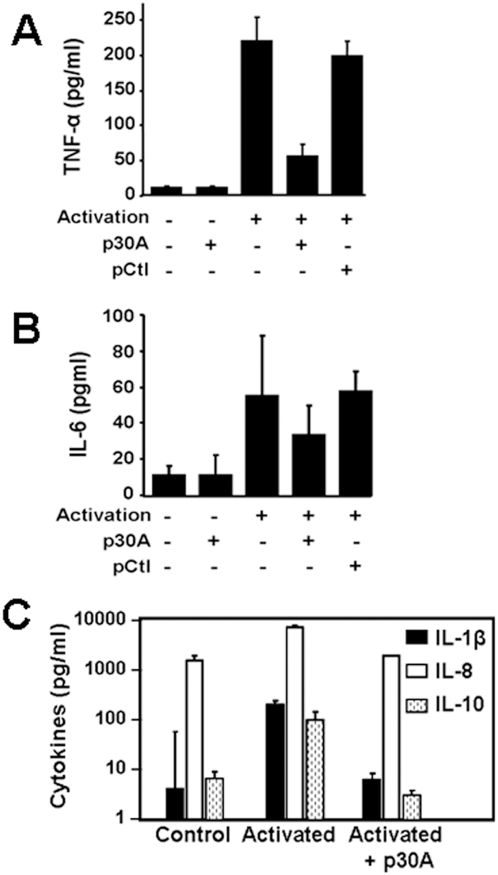
Inhibition of inflammatory mediator generation from healthy human PBL-derived macrophages by p30A. Decreased levels of TNF-α (A), IL-6 (B), IL-1β, IL-8 and IL-10 (C) were detected in CD23-activated cells after incubation with p30A. No effect was observed with control peptide (pCtl). Values are mean±s.d. of cells from 5 (IL-6, TNF-α) or 2 (IL-1β, IL-8, IL-10) different donors.

CD23 signaling mediates cell activation and the generation of various inflammatory mediators, involved in physiopathologic role attributed to this molecule [1]. Specific inflammatory gene transcription array (see Material and methods) was used to analyze the effect of p30A on CD23-mediated activation of macrophages from human healthy donors. Compared to resting CD23^+^ cells, cross-linking of CD23 induced a significant expression of 38 new mRNAs (Table 1) encoding critical inflammatory mediators, such as IL-1β, IL-6, IL-8, TNF-α, lymphotoxin-α, TNF-R1 and MIF (Figure 3 and Table 1). These findings underlined the critical role of CD23 during inflammatory response of human macrophages. Heptapeptide p30A inhibits the expression of most of the above mRNAs (24/38 were totally suppressed) including IL-1β, IL-6, IL-8, TNF-α and TNF-R1 (Figure 3 and Table I). In order to confirm these results, we further quantified TNF-α, IL-6, IL-1β, IL-8 and IL-10 at protein levels (Figure 4). Results showed that p30A significantly inhibited all CD23-mediated activation of inflammatory mediators at protein levels, supporting transcription results.

**Table 1:** mRNA expression following macrophage activation.

	Non activated cells (PBS)	Activated cells	Activated cells+p30A
**Number of expressed genes detected/114**	14	53	28
**Interleukins and their Receptors**	IL10RA, IL11RA, IL1R2, IL2RG	IFNG, IL10RA, IL10RB, IL11RA, IL13RA1, IL15RA, IL16, IL17R, IL18R1, IL1B, IL1R2, IL1RN, IL2RA, IL2RB, IL6, IL6R, IL8	IL10RA, IL10RB, IL11RA, IL17R, IL1R1, IL1R2, IL2RA, IL2RG, IL6R
**Chemokine Receptors**	CCR1, CCR5, CCR7, CXCR4	CCR1, CCR5, CCR7, CCR8, CCR9, CXCR4	CCR1, CCR5, CCR7, CXCR4
**Chemokines Subfamily A (Cys-Cys)**	CCL17, CCL25, CCL3, CCL4	CCL13, CCL15, CCL17, CCL18, CCL19, CCL2, CCL3, CCL4, CCL5, CCL7, CCL8	CCL15, CCL17, CCL18, CCL19, CCL3, CCL4, CCL5
**Chemokines Subfamily B (Cys-X-Cys)**		CX3CR1, CXCL1, CXCL10, CXCL2, CXCL3, CXCL5, CXCL9	
**Chemokines Other subfamily members**	BCL6	BCL6, BLR1, C3	BCL6, BLR1, C3
**TNF Ligands and Receptors**	LTB (LT- β)	LTA (LT-α), LTB (LT-β), TNF (TNF-α), TNFRSF1A (TNFR1), TNFRSF1B (TNFR2)	LTA (LT- α), LTB (LT-β), TNFRSF1B (TNFR2)
**Other related genes**		CEBPB, MIF, CD40LG	LTB4R, MIF

**Figure 5:**
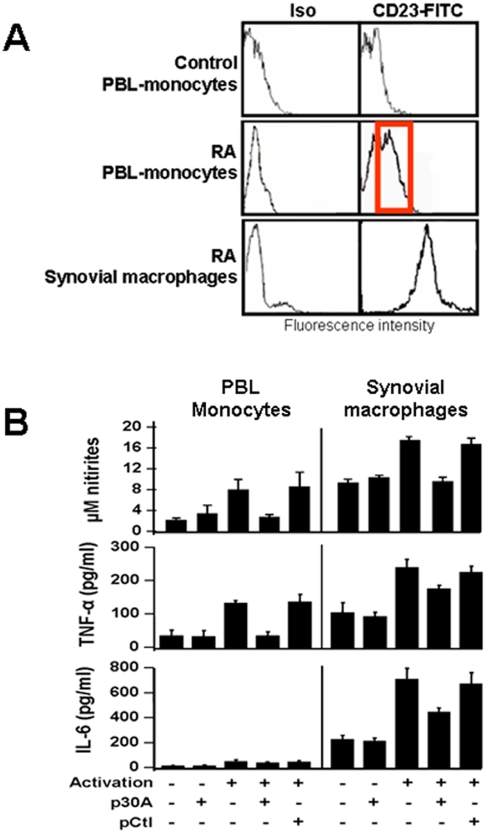
p30A inhibits the production of CD23-dependent inflammatory mediator release in cells from rheumatoid arthritis (RA) patients. (A) PBL and synovial monocytes/macrophages from RA patients were labeled with anti-CD23-FITC or isotype-matched (Iso) McAb and compared to healthy PBL. Representative fluorescence intensity data obtained from one case, out of 11 distinct patients or healthy individuals. (B) Inhibition of inflammatory mediator release from RA patients derived monocytes/macrophages. PBL monocytes or macrophages collected from synovial liquid were activated through CD23 after 1 h pre-incubation with/without p30A or pCtl. Cell supernatants were collected 72 h later and nitrites, TNF-α and IL-6 were quantified. Values are expressed as mean±s.e.m from 7 patients' cells.

To substantiate above data, p30A was tested on CD23-mediated activation of monocytes/macrophages from adult arthritic patients during rheumatoid arthritis crisis. We supported previous data showing that in those patients, high levels of CD23^+^ monocytes/macrophages are detected in their synovial fluid [19] and in peripheral blood [20], as regard to healthy individuals (Figure 5A). Further, we purified peripheral and synovial fluid monocytes/macrophages from various patients, and incubated them with cross-linking anti-CD23 McAb. Nitric oxide, TNF-α and IL-6 were quantified in culture supernatants as inflammatory markers (Figure 5B). In contrast to healthy individuals, cells from arthritic patients were shown to produce inflammatory factors following CD23 activation. As predicted, addition of p30A to cells prior to their activation reversed inflammatory mediators at the same levels than control cell-supernatants (Figure 5B). These data constitute the first evidence for direct role of CD23 in inducing inflammatory response of cells from adult arthritic patients and showed that p30A is effective in inhibiting CD23-mediated activation of monocytes/macrophages from arthritic patients.

### p30A prevents CD23-mediated activation of rat macrophages in vitro and decreases arthritic signs and bone destruction in vivo

**Figure 6:**
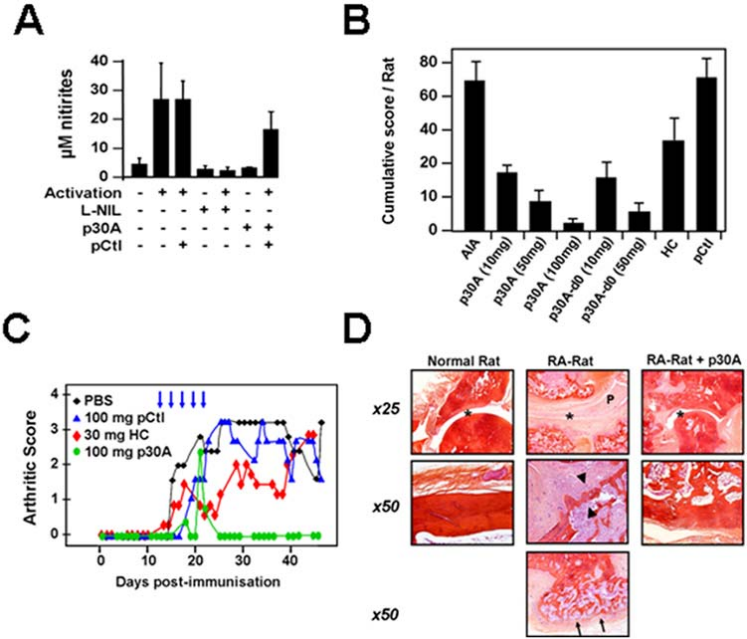
Inhibition of *in vitro* and *in vivo* inflammatory responses in rats by p30A peptide. (A) Rat peritoneal macrophages were activated *via* CD23 antigens with/without p30A, L-NIL or pCtl. Cell supernatants were collected 48 h later and nitrite levels were quantified. Values are mean±s.d. of cells from 3 different rats. (B) Cumulative clinical score/rat obtained following 50 days post-immunization in all groups. AIA rats were treated either after or before (d0) the onset of symptoms. Treatment consisted of p30A, hydrocortisone (HC, 30 mg/rat)) or pCtl (100 mg/rat). Shown are means of cumulative score/rat±s.d. from 5 (HC) or 8 rats (other conditions). (C) Evolution of arthritis severity scores of adjuvant-induced arthritis (AIA) in rats treated with intracutaneous p30A or pCtl (100 mg/rat) or hydrocortisone (after the onset of clinical signs as illustrated by arrows (means from 8 rats, s.e.m.<20%). (D) Histopathologic analysis confirm p30A ability to decrease all components of AIA lesions: pannus formation and inflammation of the synovium (P), deformity of joint and bones (asterix), bone remodeling and destruction (arrowhead), bone neoformation (arrows) and cartilage destruction.

To investigate the role of CD23 in the pathogenesis of arthritis and the effects of blocking p30A *in vivo*, we used a rat model of adjuvant-induced arthritis (AIA) which shows many clinical and histopathological features close to human rheumatoid arthritis [21]. In contrast to mice, these animals also express functional CD23 on their macrophages [22] and constitute an appropriate chronic model to study inflammatory role of both surface and soluble CD23 [21], [23]. In Figure 6A, we showed that p30A cross-reacted with CD23 and was able to reverse the IgE/DNP activation of rat macrophages *ex-vivo* as illustrated by significant decrease in CD23-mediated induction of NO generation.

**Figure 7:**
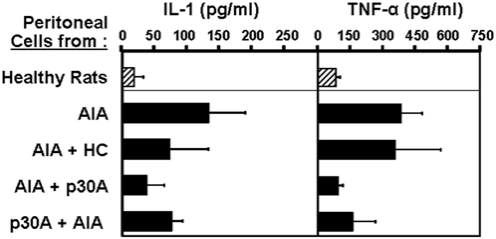
Decreased inflammatory markers of peritoneal macrophages following *in vivo* treatment with p30A peptide. AIA rats were treated either after (AIA+p30A) or before (p30A+AIA) the onset of symptoms. Treatment consisted of p30A (10 mg/rat) or hydrocortisone (HC, 30 mg/rat). Rat peritoneal macrophages were collected following 50 days post-immunization, washed and re-incubated in culture medium alone. Cell supernatants were collected 48 h later and cytokine levels were quantified. Macrophages from healthy rats were also incubated in similar conditions. Values are mean±s.d. of cells from 4 different rats.

*In vivo*, we used the same protocol as for hydrocortisone: five intracutaneous soluble doses, every two days after the onset of arthritic symptoms. To assay disease prevention potential, we injected p30A or control peptide to rats simultaneously to arthritis induction with presumed causing agents, before the appearance of any arthritic sign. As shown in Figure 6B, cumulative clinical scores obtained following 50 days post-immunization indicate that p30A significantly improved the clinical course of AIA rats as compared to the control group of non-treated rats (Figure 6B, *p*<0,0001). P30A was found to be more efficient than hydrocortisone in decreasing arthritic scores (Figure 6B, 6C) and was effective either after disease onset or for the prevention (Figure 6B, p30A-d0) of induced-disease (*p*<0.0002).. Furthermore, this beneficial effect was not found in animals treated with control peptide. Histopathologic analysis 50 days after AIA induction showed that p30A significantly decreased all components of AIA arthritic lesions: pannus formation and inflammation of the synovium (Figure 6D), deformity of joint and bones (Figure 6D asterix), bone remodeling and destruction (Figure 6D arrowhead), bone neoformation (Figure 6D arrows) and cartilage destruction. Moreover, no weight loss, neither intestinal bleeding was observed during this period (data not shown) which indicate that p30A is an effective and apparently safe therapy for AIA. To show the role of p30A on macrophage-derived mediator release *in vivo*, we have analysed the inflammatory markers of peritoneal cells from treated and non-treated AIA rats. In this model, we observed an increased IL-1 and TNFα release from peritoneal macrophages *ex vivo* following AIA development (Figure 7). After various *in vivo* treatments, peritoneal cells were isolated, promptly incubated 48 h in medium alone, and cell supernatants were analysed for the levels IL-1β and TNFα. As shown in Figure 7, AIA-derived cells expressed high IL-1β and TNFα levels compared to healthy rats. Treatment with hydrocortisone moderately decreased IL-1β levels while p30A, at both therapeutic and preventive pathways, dramatically reduced both IL-1β and TNFα levels produced by peritoneal cells *ex vivo*. Together, these results clearly demonstrate *in vivo* inhibition of immune response by p30A in AIA rats.

## Discussion

CD23 has emerged as a potential therapeutic target for a wide range of inflammatory affections [1]. As original functional approach, we here developed new specific CD23 heptapeptide (p30A) able to target CD23-mediated activation of macrophages and the subsequent secretion of pro-inflammatory mediators, including TNF-α, IL-6 and NO. Based on *in vitro* studies we showed that p30A addition to human macrophages greatly diminished the transcription of genes encoding inflammatory cytokines. This was also confirmed by significant reduction of mediator levels in cell supernatants. It was also based on *in vivo* studies that showed that administration of p30A to rat led to amelioration of adjuvant-induced chronic arthritis. We also show that p30A decreased IgE-mediated activation of both human and rat CD23^+^ macrophages. CD23-blocking peptide p30A prevents the activation of monocytes/macrophages without cell toxicity.

CD23 has two isoforms CD23a and CD23b. On B lymphocytes, CD23a is associated with endocytosis of IgE coated particles, whereas CD23b mediates phagocytosis of soluble IgE complexes and the inflammatory response in rat and human cells [1], [9]. CD23a is expressed (as by many other immune cells) by rat and human epithelial cells [5], [24] whereas in mice, CD23b is restrictively expressed. The function of CD23 is further extended by several physiological ligands. These comprise the complement receptors CD21 (CR2) [12], α_M_β_2_-integrin (CD18/CD11b) and α_X_β_2_-integrin (CD18/CD11c) [10]; the vitronectin receptor (α_V_β_3_-integrin); and α_V_β_5_-integrin [25]. Data also indicate that CD23 on epithelial cells plays a central role in transporting IgE and allergen–IgE complexes across the epithelial-cell barrier [26]. Upregulation of the CD23 has been observed during various allergic and inflammatory responses and is believed to play a role in the bronchial mucosa through the enhancement of antigen uptake and presentation [9], [27]. CD23 cross-linking mediates the activation of human macrophage inflammatory reaction in the presence of IgE. We have previously demonstrated that CD23-mediated IgE-antigen-induced activation of inducible nitric oxide synthase, IL-1 and TNF-α in various human cells including macrophages [17], eosinophils [7] and epidermal epithelial cells [5], [28]. Multiple signaling pathways, including p38 mitogen activated protein kinase (MAPK), JNK, and Erk pathways may be involved in the regulation of pro-inflammatory mediators following this CD23 activation [9]. CD23 dramatically enhanced antigen complex presentation compared to unfocused or IgG-bound antigen in animal studies. This suggests that CD23 may amplify immune response to autoantigens during arthritis.

Monocytes and eosinophils are among the different inflammation effector cells that are drawn to sites of inflammation, where IL-4, IL-13, IFN-γ and GM-CSF stimulate the expression of CD23 by these cells [7], [9]. This in turn, arms the cells for useful functions, such as clearance of antigen, killing and phagocytosis of pathogens (for example, helminthes) [17] and tumor cells that bear ‘foreign’ antigens [29]. The upregulation of CD23 also leads to the release of soluble fragments that bind to integrins on monocytes, and thereby initiate the production of tumor-necrosis factor and other pro-inflammatory cytokines [30]. In carrying out their functions, monocytes and eosinophils inevitably also inflict some damage on bystander cells in the tissue. This side effect ultimately contributes to tissue remodeling and exacerbates the symptoms of various inflammatory diseases [7].

Authors reported overexpression of membrane bound and sCD23 during acute juvenile or adult arthritis, with a clear pro-inflammatory property in this form [15]. In the present study, we report similar observations in peripheral and synovial liquid monocytes/macrophages from adult arthritic patients (Figure 5). We also showed the capacity of these CD23^+^ cells to directly undergo CD23 activation, in contrast to cells from healthy individuals. These results reinforced the notion of the systemic involvement via the peripheral monocytes in RA diseases and pointed out the role of CD23 as disease-related marker during arthritic crisis. Steroids have a clear inhibitory effect on CD23 expression and cell signaling, which may account for their anti-inflammatory effects [31]. Moreover, CD23-mediated signaling and subsequent nitric oxide and TNF-α production by human macrophages and epithelial cells were also been shown to be decreased by IL-10 [32], a well-known immunoregulator and Th-1 inhibitor cytokine.

CD23 was also directly triggered by specific antibodies as therapeutic approach. This was firstly showed in an experimental arthritic mice model where an anti-CD23 administration decreased all pathological signs and corroborated studies showing a reduced arthritic signs in CD23-deficient animals [13], [14]. Primate/human CD23-McAb, lumiliximab, developed for the treatment of chronic lymphocytic leukemia, has also been shown to a promising therapy in Phase I of clinical trials for asthma [9]. Similarly, p30A specifically bound human and rat CD23, blocked CD23-mediated cell activation and decreased CD23-mediated activation of peripheral and synovial monocytes/macrophages from patients during arthritic crisis. To confirm these results, we used rat model of chronic arthritis because, as human cells, rat macrophages are known to fully express CD23b isoform *in vivo* [22] in opposition with mouse model. Clearly, p30A decreased arthritic signs in rats in a dose-dependent manner. Furthermore, administration of p30A prior to disease induction prevented the development of severe clinical signs in rats. These data clearly support a role for CD23 in the pathogenesis of arthritis and the interest of the use of small peptides as therapeutic tools [33]. Data in rat model of chronic arthritis show a clear dose-dependent long term effect (>one month), indirectly supporting the stability of p30A *in vivo*. P30A had several advantages: small size, easy synthesis and the absence of side agonistic effect compared to the pro-apoptotic activity of anti-CD23 antibodies [34]. Even if further investigations are needed to confirm these results in human, p30A should be considered as a potent candidate molecule in the treatment of RA.

## Materials and Methods

### Anti-CD23 peptides

p30A peptide used in this study is a synthetic linear heptapeptide (FHENWPS) obtained by panning a phage displayed peptide M13 library (Ph.D 7, New England BioLabs, Ipswich, MA, USA) on purified sCD23 protein (soluble 25 kDa, kind gift from E. Kilcchher, Novartis, Basel) [35] as detailed elsewhere (PCT Patent 098435). As control (pCtl), we have used synthetic heptapeptide with unrelated sequence (SFNYNYA).

### Human Cells

Peripheral blood samples pre-tested for the absence of HIV or hepatitis virus infections were obtained from healthy volunteers (EFS Aquitaine, Bordeaux Blood Bank). We also collected paired peripheral blood and synovial fluid from seven patients, who fulfilled the American College of Rheumatology criteria for RA, during arthritic crisis. Written informed consent was obtained from all the patients. This study was carried out in compliance with the Helsinski Declaration and was approved by the National Ethical Authorities. All seven patients had previously received conventional therapy but none has been given TNF blocker or any other biologic treatment for their condition. Whole leukocytes were then suspended in McCoy 5A modified culture medium supplemented with 100 U/ml penicillin, 100 µg/ml streptomycin, 25 mM HEPES, 0.1 mM 2-mercaptoethanol, 2 mM sodium pyruvate, 0.2 mM L-cysteine, 5 µg/ml polymyxin B and 10% fetal calf serum (FCS) (all from Gibco-Europe, Paisley, Scotland). All the above culture medium, chemicals, and FCS were endotoxin-free and tested for the absence of direct activation effects on human monocytes (CD23 expression and TNF-α production as activation markers). Peripheral blood-derived mononuclear leukocytes (PBL) were obtained by Ficoll gradient separation and monocytes were subsequently separated from other leukocytes by adherence to FCS-coated culture flasks or CD14 beads (Miltenyi Biotec, Paris, France). Synovial fluid was diluted with culture medium and adherent cells obtained following incubation into FCS-coated culture flasks. Following these procedures, >95% of cells expressed CD14 antigen and displayed cytochemical characteristics of monocytes/macrophages [17].

### Human cell activation

Monocytes or macrophages were activated through CD23 pathway: while monocytes/macrophages from RA patients express CD23, monocytes from healthy donors must be pre-activated to express surface CD23 as described [17]. This pre-activation was obtained by incubation of healthy monocytes with recombinant human IL-4 (20 ng/ml) during 48 h at 37°C; After washing, all cell populations were tested by flow cytometry for their surface CD23 expression (>80% CD23^+^) and then incubated in the presence of cross-linking CD23-MAb (clones 135 or DM2, IgG1k, 20 µg/ml) during 48–72 h, or IgE and anti-IgE as described [5], [17]. When indicated, peptides were added to the cells 2 h prior to their activation. Cells supernatants were then analyzed for their nitric oxide content (see below) and for the presence of cytokines: levels of TNF-α and IL-6 were determined in duplicates for each sample by a specific ELISA (R&D, Paris, France) while levels of IL-1β, IL-8 and IL-10 was determined using FlowCytomix Human Th1/Th2 Kit as recommended (Bender Medsystems, Vienna, Austria).

### Western blot

Total cellular proteins were isolated from CD23^+^, ADAM B-cell line in cell lysis buffer (10 mM Tris pH 8.0, 150 mM NaCl, 5 mM EDTA, 1% Triton X-100, 0.05% SDS, 10 mM Sodium Fluoride, 1 mM Sodium Orthovanadate, 5 µM Molibdic Acid) and a mixture of protease inhibitors (all from Sigma Aldrich, Saint Quentin Fallavier, France). Protein concentration was determined using the Bradford assay (Bio Rad, Hemel Hempstead, UK). Protein samples (50 µg total protein) were subjected to SDS-PAGE using a 12% resolving gel. The separated proteins were electro-transferred to PDVF membrane (Millipore, Bedford, UK) for 1 h to 15 V. The membrane was blocked 1 h at room temperature in 5% BSA in TBS-tween (TBS-T) and incubated with different concentrations of p30A (10, 20 and 40 µg/ml) in TBS-T for 2 h. The membranes were then incubated with either 0.5 µg/ml mouse anti-human CD23 or 0.5 µg/ml mouse anti-human Bcl2 (both from Dako Cytomation, Glostrup, Denmark) overnight at room temperature. Membranes were washed 18 h later in TBS-T and re-incubated with goat anti-mouse IgG peroxydase-conjugated (1/10000, Vector, Burlingame, USA) for 1 h at room temperature. The membranes were washed in TBS-T and visualized with an enhanced chemiluminescence system (Perkin Elmer, Boston, USA). Immunoreactive bands (CD23 and Bcl2) were detected using the FluorChem 8800 (Alpha Innotech, San Leandro, USA) and semi-quantified by densitometry using AlphaEase FC (Alpha Innotech).

### RNA preparations and transcriptomic arrays

Following cultures, total RNA was extracted using RNeasy kit (Qiagen, Hilden, Germany). Synthesis, purification and hybridization of biotin-labeled cRNA (6 µg) to custom array membranes were performed according to manufacturer's recommendations (Oligo GEArray® Human Inflammatory Cytokines and Receptors Microarray, ref OHS-011, Superarray Bioscience, Frederick, MD). After local background subtraction, average signal intensity from duplicate spots was compared to values obtained for housekeeping genes using AlphaImager HP automatic image capture software (Alpha-Innotec, San Leandro, CA). For each gene, modulation was defined as the relative expression value for stimulated *vs.* control sample.

### Analysis of NO pathway

Culture supernatants (72 h) were assayed for the stable end-product of NO, NO_2_^−^ using the Griess reaction modified as detailed elsewhere [36]. Inhibitory analog of L-arginine, L-NIL (N(6)-(1-iminoethyl)-L-lysine/2HCl, Coger SA, Paris, France) was used to inhibit iNOS-mediated NO generation. This method gave a sensitivity limit of 0.2 µM if low NO_2_^−^ medium (DMEM) was used. Total RNA was isolated as above from peritoneal macrophages. Reverse transcription-PCR was performed with an automatic thermal cycler (iCycler, Biorad) using the following specific primers: iNOS mRNA sense (5′-ATGCCAGATGGCAGCATCAGA-3′, exon 8) and iNOS mRNA antisense (5′-ACTTCCTCCAGGATGTTGTA-3′, exon 11). Hypoxanthine phosphoribosyltransferase (HPRT) mRNA sense (5′-TATGGACAGGACTGAACGTCTTGC-3′) and HPRT mRNA antisense (5′-GACACAAACATGATTCAAATCCCTGA- 3′) primers were used as controls. The iNOS messenger is represented by a 371-bp band, whereas a 496-bp band indicates the HPRT messenger [17].

### Experimental arthritis and rats

All animal procedures were performed in strict accordance with the guidelines issued by the European Economic Community “86/609”. Female Lewis rats (Janvier, Le Genest St Isle, France) were housed under standard laboratory conditions with free access to food and water. The temperature was kept at 22±2°C and a 12 hour light/dark schedule was maintained.

For macrophage collection, animals were anaesthetized with ether and the peritoneal cavity was washed with 10 ml of cold PBS, pH 7.4. After centrifugation at 1200 g for 10 min at 4°C, cells were collected, counted and adjusted to 2×10^5^ cells per ml with RPMI 1640 culture medium supplemented with 100 U/ml penicillin, 100 µg/ml streptomycin and 10% heat-inactivated FCS. To activate the production of various inflammatory mediators from rat peritoneal macrophages, cells were incubated with lipopolysaccharide (LPS, 1–5 µg/ml; Sigma), or IgE/DNP as described [19]. Peptides were added one hour prior to cell activation. Cells or their culture supernatants were then tested for the presence of various inflammatory mediators.

Adjuvant arthritis was induced in six week-old animals by subcutaneous injection at the base of the tail of 300 µl containing 1.8 mg of inactivated *Mycobacterium butyricum* (Difco Laboratories, Detroit, MI) diluted in emulsion of 8 ml Vaseline, 1 ml polysorbate 80, and 1 ml PBS (Phosphate Buffer Saline, Eurobio). Rats were boosted one week later following the same procedure. Observation was conducted for up to 50 days following immunization for clinical signs of arthritis. Rats were divided in different groups of 8 rats. Groups were treated with five injections (every 2 days) of either p30A (10, 50 or 100 mg in a PBS solution), pCtl, hydrocortisone (Sigma Aldrich) or PBS solution. Injections were initiated two days after the appearance of first arthritic symptoms (therapeutic) or on the first day of immunization (preventive). Evaluation of AIA severity was performed by two independent observers who where not aware of the study protocol. The severity of AIA in each paw was quantified daily using an ordinal clinical score measurement from 0 to 4 as follows: no signs of inflammation ( = 0); swelling alone (>2 fold paw diameter) ( = 1) or swelling/immobility ( = 2) of one paw; swelling ( = 3) or swelling/immobility ( = 4) of two paws. Weight evolution of rats was measured daily. Rats were sacrificed 50 days after AIA induction. Peritoneal macrophages were collected and incubated in culture medium alone for 48 h. Quantification of TNF-α and IL-1β in rat cell supernatants was performed using appropriate ELISA kits and according to manufacturer's recommendations (Biosource, Montrouge, France).

For histological analysis, the forelimbs were fixed in 4% buffered formalin, decalcified by electrophoresis in decalcifying buffer (Bayer Diagnostic, Puteau, France) for 3 h and embedded in paraffin. Thick sections of five µm were stained with hematoxylin, eosin, safran (HES) using routine protocols.

### Statistical analysis

Comparisons were assessed using Fischer's exact test for proportions and Mann-Whitney-U test for quantitative values. *P*<0.05 was considered to be significant. Results were analyzed and compared using the Student *t*-test for paired data.
